# Evaluation of good review practices in member authorities of the East African Medicines Regulatory Harmonisation initiative: strategies for alignment with African medicines agency

**DOI:** 10.3389/fmed.2024.1437970

**Published:** 2024-08-29

**Authors:** Nancy Ngum, Margareth Ndomondo-Sigonda, Rémy Habonimana, Fred Siyoi, Clarisse Irasabwa, Julia Ojukwu, Felchism Apolinary, Andrew Okello, Sabrina Ahmada, Stuart Walker, Sam Salek

**Affiliations:** ^1^Department of Clinical and Pharmaceutical Sciences, School of Life and Medical Sciences, University of Hertfordshire, Hatfield, United Kingdom; ^2^African Union Development Agency – New Partnership for Africa’s Development (AUDA-NEPAD), Johannesburg, South Africa; ^3^Burundi Food and Medicines Regulatory Authority (ABREMA), Bujumbura, Burundi; ^4^Pharmacy and Poisons Board-PPB, Nairobi, Kenya; ^5^Rwanda Food and Drugs Authority, Kigali, Rwanda; ^6^Drug and Food Control Authority (DFCA), Juba, South Sudan; ^7^The Tanzania Medicines and Medical Devices Authority (TMDA), Dodoma, Tanzania; ^8^National Drug Authority, Kampala, Uganda; ^9^Zanzibar Food and Drugs Authority (ZFDA), Dodoma, Tanzania; ^10^Centre for Innovation in Regulatory Science, London, United Kingdom; ^11^Institute of Medicines Development, London, United Kingdom

**Keywords:** East African Medicines Regulatory Harmonisation (EAC-MRH), good assessment procedure, good review practices, regulatory reliance, African Medicines Agency (AMA)

## Abstract

**Introduction:**

The East African Community Medicines Regulatory Harmonisation (EAC-MRH) programme was established to address challenges faced by national regulatory authorities (NRAs) of the region. Work sharing through joint assessments and inspections was adopted to manage limited resources and capacity; however, NRA good review practices (GrevP) are also a key determinant to success. This study evaluated GReVP among the EAC-MRH NRAs and mapped required strategies for countries to align themselves with the African Medicines Agency (AMA).

**Methods:**

A validated questionnaire (Optimising Efficiency in Regulatory Agency—OpERA) that standardises and captures review processes was completed by the head of the medicines registration division in each NRA. A country report based on the completed questionnaire was developed for each NRA and validated by the heads of the respective authorities.

**Results:**

The population and size of the NRAs vary and four of the countries have semi-autonomous authorities and three NRAs are autonomous. The Burundi and South Sudan authorities were fully government funded, Kenya and Uganda entirely from fees, while Rwanda, Tanzania and Zanzibar were partially funded from different sources. All authorities except South Sudan, which does not receive or review applications had backlogs. Authority fees varied based on the different application categories. Key milestones for standardised regulatory processes are implemented in all authorities. Queue times range from a few weeks to about one year. Three NRAs use internal technical agency staff for scientific assessments and three use both internal and external experts. Clock stop time varies and target timelines for review committee range from one day to three months. All the NRAs implement some best practices on quality measures, transparency and communication. Some have activities for transparency improvement but with minimal attention to training and education. Most employ some quality decision-making practices.

**Discussion:**

GrevP in EAC-MRH NRAs still needs to be improved and it is imperative that these authorities streamline and harmonise their practices. Increasing human resources and an investment in training and education of staff will enable the implementation of all measures for GRevP. This is vital, as the effectiveness and efficiency of the AMA will depend on the strength of these NRAs.

## 1 Introduction

The East African Community (EAC) is made up of seven countries: the Republics of Kenya, Uganda, Rwanda, Burundi, South Sudan, the Democratic Republic of Congo (DRC) and the United Republic of Tanzania. The DRC was admitted in 2022, after this study had been conducted. This intergovernmental organisation with a population of 303,397,152 has its headquarters in Arusha, Tanzania.

The countries in this region have common medicines regulatory challenges such as differences in laws and regulations and the inadequate capacity of the national medicines regulatory authorities (NRAs) (1). To address these challenges, the EAC Secretariat, in collaboration with the EAC NRAs, established the East Africa Medicine Harmonisation (EAC-MRH) project in 2012 as the regional coordinating body of the African Medicines Harmonisation (AMRH) initiative. This project was part of the implementation of one of the provisions of the EAC Treaty, Chapter 21, Article 118 on regional harmonisation in health captured in the EAC Compendium, 2014.

### 1.1 Operational aspects of EAC-MRH

The EAC-MRH is one of the five regional medicines regulatory harmonisation programmes in Africa. There are seven NRAs of the region participating in the EAC-MRH initiative, representing countries that share a common history, market, language, culture, and that already had a treaty in place that called for harmonisation. Since its inception, the aim of the programme has been to reduce registration timelines for medical products through joint reviews and inspections, with an overall goal to enhance access to safe, efficacious and quality medicines by patients in the region. Using harmonisation and work sharing, the EAC-MRH has conducted 25 joint assessments in approximately 10 years, with about 202 products reviewed and 107 recommended for registration by the EAC partner states (2). However, due to the long bureaucratic process for the review and approval of the official notification letters to applicants, the median time for the communication of approval to the applicant following the scientific assessment generally exceeded the EAC target of 30 calendar days (1). A key challenge faced in this work-sharing initiative is delay in granting marketing authorisation (MA) by the NRAs, reflecting the varying timelines for products to be registered at the national level after a regional recommendation is made (2).

According to Mashingia et al. (1), the EAC target time for granting a MA of 116 calendar days was far exceeded by all five authorities. The median times for granting MA by Burundi (ABREMA), Kenya (PPB), Rwanda FDA, Uganda (NDA), and Tanzania (TMDA) were 965, 683, 649, 582, and 515 calendar days, respectively. Several reasons have caused the long median times to grant the MA by the EAC NRAs, including lengthy administrative procedures, such as NRA requirements for product applications to be considered first by the scientific committee before issuance of an MA certificate; delays by applicants in paying fees for registration after filing for MA in NRAs; and differing maturity levels and limited capacities and capabilities to conduct timely scientific reviews among NRAs, with applicants expected to pay varying amounts for NRA fees (Table 1).

**Table 1:** Size of authorities.

Measure	Burundi	Kenya	Rwanda	South Sudan	Tanzania	Uganda	Zanzibar
Population (millions)	13.1	54.9	13.2	11.3	65.4	45.7	1.7
Authority staff	32	170	188	42	336	292	150
Number of internal reviewers	4	28	15	4	45	33	12
Reviewers in authority staff	12, 5	16%	8%	10%	13%	11%	8%
Total applications received	70	997	659	0	858	861	10
Number of applications per reviewer	23	36	44	0	19	26	1

This study is therefore aimed to evaluate good review practices (GReVP) in the authorities participating in the EAC-MRH initiative and map strategies for moving forward as they go through the process of alignment with the operationalisation of the African Medicines Agency (AMA).

This is the first in a two-part series, with the second article focussing on the review models and timelines of these regulatory authorities.

## 2 Materials and study participants

### 2.1 Study participants

The study participants included senior programme officers heading the medicines registration divisions in the seven NRAs: Pharmacy and Poisons Board (PPB), Kenya; National Drug Authority (NDA), Uganda; The Tanzania Medical Devices Authority (TMDA); Zanzibar Food and Drugs Authority (ZFDA) Tanzania; Drug and Food Control Authority (DFCA) South Sudan; Burundi Food and Medicines Regulatory Authority (ABREMA) and the Rwanda Food and Drugs Authority.

### 2.2 Data collection

**Figure 1:**
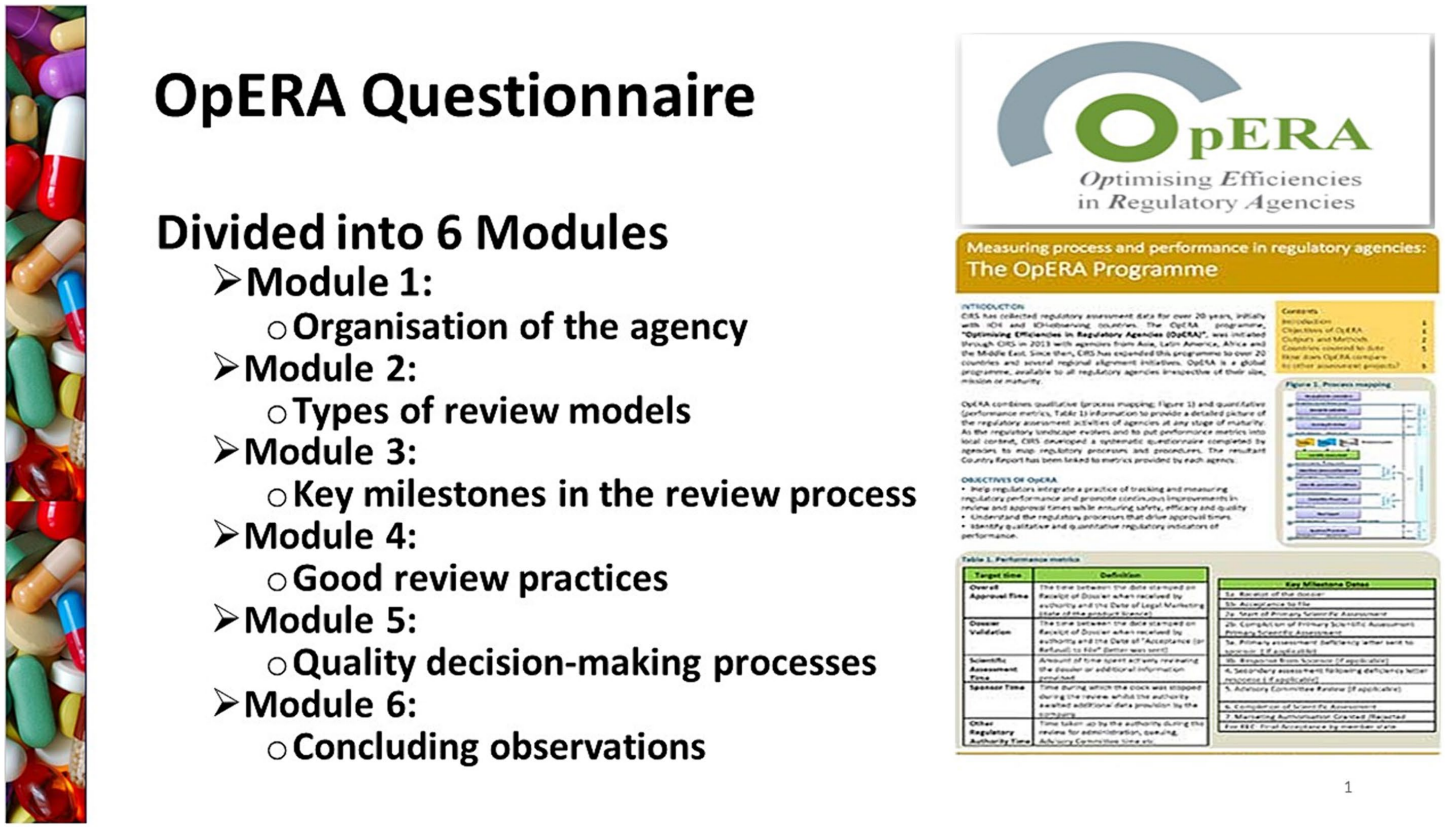
A validated questionnaire (Optimising Efficiency in Regulatory Agency - OpERA) describing the organisation, structures and regulatory review systems.

A validated questionnaire (Optimising Efficiency in Regulatory Agency—OpERA) describing the organisation structures and regulatory review systems for market authorisation of new active substances (NASs) and generics, including their overall timelines from the date of submission of the application to when it is approved, GRevP and quality decision-making practices, was completed by each of the authorities in 2022 (Figure 1). The full OpERA Questionnaire is provided as Supplementary material.

The questionnaire was composed of six different parts: Part 1—Organisation of the authorities with focus on its structure and resources; Part 2—types of review models used by the authorities for scientific assessment of medicines; Part 3—key milestones in the review process with focus on the process map and milestones; Part 4—GReVP and how the authorities build quality into their regulatory processes; Part 5—quality of the decision-making processes based on whether the authorities have good measures in place to guide decision making; and Part 6—concluding observations that relate to the strengths and challenges for the authorities to carry out their mandates.

## 3 Results

For the purpose clarity, the results of this first article of the series will be presented in four parts: Part 1—Organisation of the regulatory authorities; Part II—Key Milestones in the review process; Part III—Good Review Practices; Part IV—Quality Decision-Making Practices.

### 3.1 Part 1: organisation of the regulatory authorities

The population and size of the regulatory agency of the six countries in the region vary (Table 1). The top two countries with the largest population are Tanzania (65.4 million) and Kenya (54.9 million). Four countries (Kenya, Rwanda, Burundi, Zanzibar), have semi-autonomous authorities and operate within the administrative structure of their Health Ministries, while South Sudan, Uganda and Tanzania have autonomous authorities independent from their Ministries of Health. Six of the authorities regulate medicinal products, medical devices, and *in vitro* diagnostics for human and veterinary use. Only the Burundian authority regulates food and medicines for human but not veterinary use.

Most of the staff in the seven authorities were pharmacists Kenya had the highest proportion of reviewers to total agency staff (16%) followed by Tanzania (13%), Burundi (12.5%), Uganda (11%), South Sudan (10%), Rwanda (8%), and Zanzibar (8%). Only Tanzania indicated they used external experts for review of applications for marketing authorisation (Table 1).

If all applications received in 2022 were reviewed, then the number of applications reviewed per reviewer in each of the authorities would be 44 applications by Rwanda FDA, 36 in Kenya PPB, 26 by Uganda, 23 in Burundi (ABREMA), 19 in Tanzania (TMDA) 1 by Zanzibar, and 0 by South Sudan (DFCA). However, all the six authorities apart from South Sudan, which does not receive, or review applications, indicated they had backlogs. Therefore, not all the applications received for that year were reviewed within the same period.

#### 3.1.1 Source of funding

The Burundi and South Sudan authorities were fully funded by their governments. The source of funding for Kenya and Uganda agency was reported as entirely from fees, while Rwanda, Tanzania and Zanzibar were partially funded from different sources. For Rwanda 22% came from the government, 76% from fees and 2% donations from partners. For Tanzania, 11.7% government; 76.3% fees; 0.6% development partners and 11.4% balance from previous budget. For Zanzibar, the government provides 49.6%, fees 41.6% and donors 8.8%. The fees charged by each agency varied between $500 and $1,000 to $2,000, based on the different kinds of application categories received (new chemical substances, biologicals, and generics). Kenya charged the lowest fees ($500) for local manufacturers for all categories, while Tanzania charged the highest fees ($3,500) for review of biologicals. Burundi and South Sudan authorities do not charge fees for applications for marketing as they are fully funded by government. The Burundi agency however charges fees for some activities such as registration and importation and these fees are put into the national bank and not in the authority budget. Each year, the Burundi government then gives the authority a fixed budget for operating costs (Table 2). Generally, authorities that fully depend on the government as their main source of funding charge fewer or lower fees, compared with authorities that are fully reliant on fees.

**Table 2:** Comparison of the fees charged (USD) and source of funding in 2023.

Measure	Burundi	Kenya	Rwanda	South Sudan	Tanzania	Uganda	Zanzibar
Source of funding	Government, 100%	Fees, 100%	Partially funded from different sources: Government, 22% Fees, 76% Donations from partners, 2%	Government, 100%	Partially funded from different sources: Government, 11.7% Fees, 76.3% Development partners, 0.6% Balance from previous budget, 11.4%	Fees, 100%	Partially funded from different sources: Government, 49.6% Fees, 41.6% Donors, 8.8%
Total annual budget	400-600,000,000 Burundi francs	US $13,796,120	US $9,155,400	8,000,000 million South Sudanese pounds, 2019–2020	US $19,123,740	US $603,554	US $826,483 (2023)
Fees for review of a new chemical entity (USD)	N/A	International, $1,000 Local$, $500		N/A	$2,000	$2,000	N/A
Fees for review of biologicals (USD)	N/A	International, $1,000 Local, $500	1,250	N/A	$3,500	$2,000	$2,000
Fees for review of generics (USD)	N/A	International, $1,000 Local, $500	1,250	N/A	$2,000	$2,000	$1,000

### 3.2 Part II: key milestones in the review process

**Figure 2:**
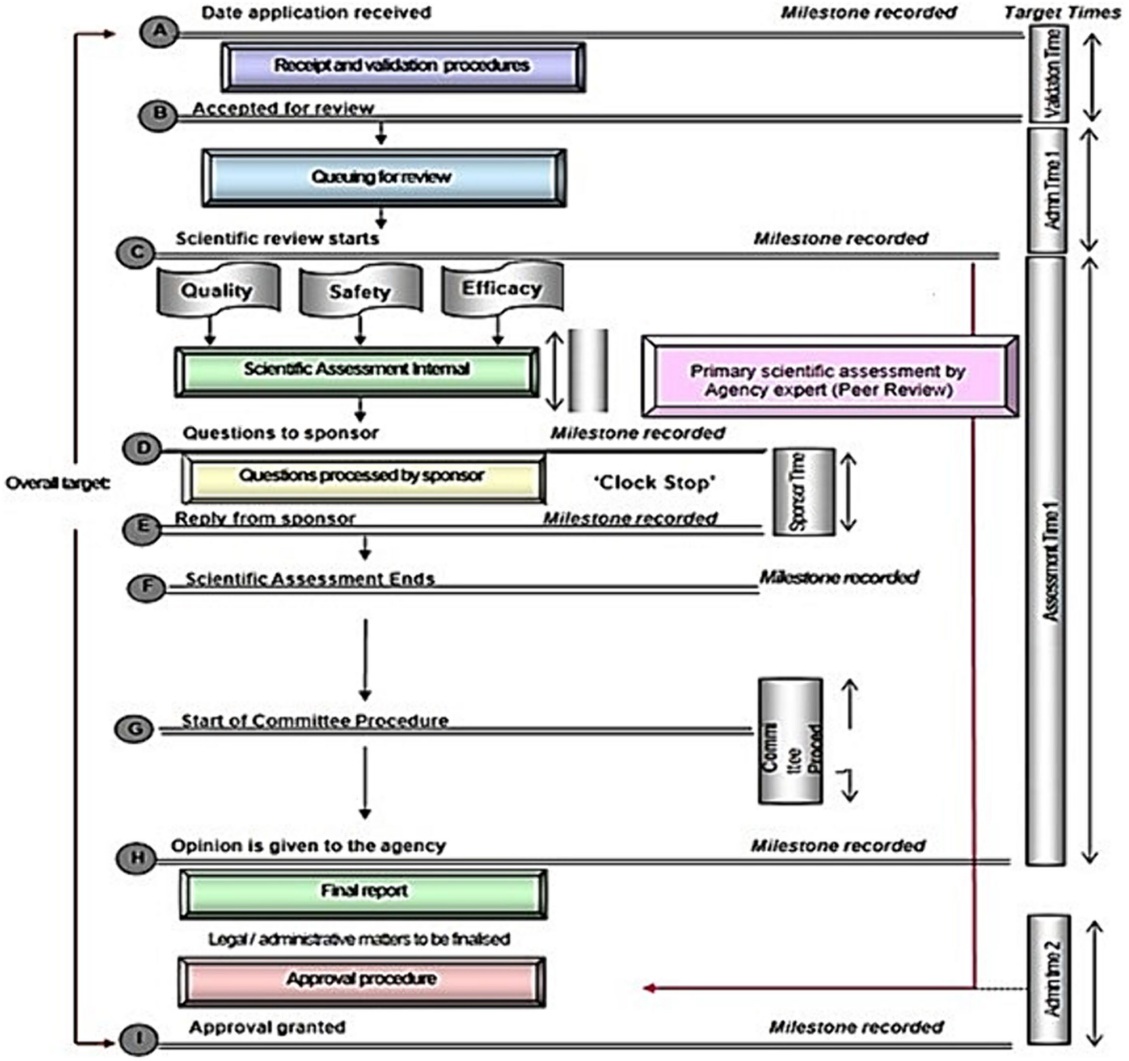
Standardised process map for the review and approval of medical products as the output from the OpERA questionnaire [adopted from Sithole et al. (3)].

Figure 2 shows a standardised review process map implemented in well-resourced regulatory systems, with key milestones recorded after each phase. This process map is a simplified version of the key steps taken during the review of an NAS and does not include rejections. The focus here is mostly on products that only go through one cycle of review, although it usually will take more than one cycle for most applications to be reviewed and a recommendation made. South Sudan will not be part of the analysis in this section as DFCA is yet to engage in review activities as key points in the review procedure and timelines are not applicable or cannot be confirmed.

#### 3.2.1 Receipt and validation procedure

All authorities indicated that when the application is received, they begin by checking for correctness. The applicant is notified if the application is incomplete and given a time limit to respond that varies across the authorities. If this timeline is not respected, then the application is considered as withdrawn. Items checked at this stage may include the legal status of the applicant or local agent; the good manufacturing process (GMP) status of the manufacturer; proof that correct fees have been paid; acceptable format, which could include International Council for Harmonisation of Technical Requirements for Pharmaceuticals for Human Use (ICH), common technical document (CTD) or local requirements and correct sections of scientific data. It is at this point where the authorities decide the kind of review pathway that will be conducted (full review, abridged or verification). Successful applications are then placed in the queue for scientific assessments.

#### 3.2.2 Queue time

After completion of the validation process, the queue time between validation and start of primary scientific assessment commences. All authorities recorded this milestone, but implemented different queue times, ranging from a few weeks in some authorities to about one year in others: Tanzania, 2–8 weeks, Burundi, Rwanda, 2–6 months, Zanzibar, 60–180 days, Uganda, 12 months, and Kenya, more than one year. Priority products are not included in the queuing system.

#### 3.2.3 Primary scientific assessment

Milestone 3 is the start of the scientific assessment, which was recorded by six authorities. Rwanda, Zanzibar and Burundi use internal technical agency staff for scientific assessments, while Tanzania, Kenya, and Uganda use both internal and external experts for the primary scientific assessment and detailed assessment report, recommendations and clinical opinion. Rwanda, Zanzibar, Tanzania and Kenya indicated that scientific data are categorised into quality, safety and efficacy; Burundi and Uganda do not separate these data although quality, safety and efficacy are reviewed in this sequence.

The term “primary assessment” is used to indicate that the internal reviewers initially evaluate the applicants’ submission in terms of the quality, safety and efficacy, which are reviewed sequentially (Figure 2). In order to clarify the situation, it should be emphasised that checking for completeness and availability of full documentation is a process of “validation,” as noted in Figure 2. The “primary scientific assessment” is carried out by the internal reviewers [who have been trained for the review process as well as seconded to other mature (maturity level 3/4) authorities]. The internal reviewers evaluate the full documentation including chemistry, manufacturing and controls (CMC) and preclinical data, as well as the totality of the clinical development from which they draw recommendations and conclusions. It should be noted that not all reviewers would engage in all such assessments, rather each reviewer would assess the area for which they are trained. In addition, it is the responsibility of the internal reviewers to provide questions arising from their assessment to the applicants and then assess their responses. Subsequently these evaluations are presented to the “scientific committee” which is often referred to as “peer review.”

#### 3.2.4 Questions to applicants

Six authorities indicated that no meetings can be held by sponsors with the staff to discuss any queries emanating from the assessment. Rather, the questions are consolidated into a single batch and sent to the sponsor. At this stage, the clock stops for Kenya, Burundi, Zanzibar and Tanzania as the applicant is given time to respond. The clock stop time varies among authorities. Uganda and Rwanda do not stop the clock while questions are being answered by the applicant.

#### 3.2.5 Review by expert committees

The principles on which the Scientific Committee operate underpin the breadth and depth of their peer review. The Scientific Committee, sometime known as the Registration Committee, consists of both internal and where appropriate, external expert regulatory reviewers. They have received a full assessment report from the internal reviewers as well as itemised responses from the applicant together with their recommendation for approval or rejection. In certain cases, this is supplemented by a proposed “risk assessment plan” for the respective product.

Five of the authorities engage a committee of experts in the review process. These experts are consulted after the authority has reviewed and reported on the scientific data. Target timelines for the start and finish for the committee vary from one day (Tanzania) to one month (Uganda) to three months (Burundi and Zanzibar). Kenya does not have a target timeline for the committee. The report from the committee is presented to the board in most of the authorities for review. In some of the authorities (Burundi, Rwanda) they are mandated to follow the committee’s recommendations, but other authorities are not mandated to do so (Uganda, Kenya, Tanzania).

#### 3.2.6 Authorisation procedure

Three of the NRAs (Kenya, Zanzibar and Uganda) inform their sponsors of a positive scientific opinion before the authorisation is issued, while the other three NRAs (Burundi, Tanzania and Rwanda) do not.

### 3.3 Part III: good review practices

#### 3.3.1 Quality measures

A comparison of the quality measures implemented by the seven regulatory authorities is illustrated in Table 3. Burundi, Kenya, Rwanda, Tanzania and Uganda implemented all eight quality measures. All of these five authorities good review practices as well as having a dedicated quality department and except for Zanzibar also employed a peer review committee. All six NRAs participated in shared and joint reviews. South Sudan did not implement any of the measures, possibly because they are not reviewing any products currently.

**Table 3:** Comparison of the quality measures implemented by the authorities.

Quality measure				Regulatory authority			
	Burundi	Kenya	Rwanda	South Sudan	Tanzania	Uganda	Zanzibar
Good review practice system	✓	✓	✓	x	✓	✓	✓
Internal quality policy	✓	✓	✓	x	✓	✓	✓
Standard operating procedures for guidance of assessors	✓	✓	✓	x	✓	✓	✓
Assessment templates	✓	✓	✓	x	✓	✓	✓
Peer review (internal)	✓	✓	✓	x	✓	✓	
Dedicated quality department	✓	✓	✓	x	✓	✓	✓
Scientific Committee	✓	✓	✓	x	✓	✓	✓
Shared and joint reviews	✓	✓	✓	x	✓	✓	✓

x, not implemented; ✓, formally implemented.

#### 3.3.2 Transparency and communication

In assessing the implementation of nine best practices in transparency and communication (Table 4), six authorities reported that they have in place official guidelines to assist industry and a list of approved products that allow for industry to track progress of their applications via email and telephone. Three authorities do not provide post-approval feedback to applicants on the quality of the submitted dossiers. Only three authorities (Burundi, Rwanda and Uganda) provided details of technical staff to contact during the review of applications and only one country (Uganda) publishes the advisory committee meeting dates. Four authorities, namely Kenya, Uganda, Zanzibar and Tanzania reported that they do publish summary of assessment reports on which the approval was granted.

**Table 4:** Comparison of the transparency and communication parameters in the authorities.

Quality measure				Regulatory authority			
	Burundi	Kenya	Rwanda	South Sudan	Tanzania	Uganda	Zanzibar
Post-approval feedback to applicant on quality of submitted dossiers	✓	✓	x	x	x	✓	✓
Details of technical staff to contact	✓	x	✓	x	x	✓	x
Pre-submission scientific advice to industry	✓a	✓	✓	x	x	✓	x
Official guidelines to assist industry	✓	✓	✓	x	✓	✓	✓
Industry can track progress of applications	✓	✓	✓	x	✓	✓	✓
Publication of summary of grounds on which approval was granted	X	✓	x	x	✓	✓	✓
Approval times	✓	✓	✓	x	✓	✓	✓
Advisory committee meeting dates	x	x	x	x	x	✓	x
Approval of products	✓	✓	✓	x	✓	✓	✓

x, not implemented; ✓, formally implemented; ✓a, informally implemented.

#### 3.3.3 Continuous improvement initiatives

Five areas (external and internal quality audits; internal tracking systems, reviews of assessors’ and stakeholders’ feedback), were assessed to determine continuous improvement initiatives in six regulatory authorities (Table 5). Tanzania implemented all five initiatives, while Burundi, Uganda, Kenya and Zanzibar implemented four out of the five initiatives. Rwanda implemented three.

**Table 5:** Comparison of continuous improvement initiatives in the authorities.

Quality measure	Regulatory authority						
	Burundi	Kenya	Rwanda	South Sudan	Tanzania	Uganda	Zanzibar
External quality audits	x	x	x	x	✓	x	x
Internal quality audits	✓	✓	✓	x	✓	✓	✓
Internal tracking systems	✓	✓	x	x	✓	✓	✓
Reviews of assessors’ feedback	✓	✓	✓	x	✓	✓	✓
Reviews of stakeholders’ feedback	✓	✓	✓	x	✓	✓	✓

#### 3.3.4 Training and education

Measures that were assessed that contribute to the development of staff and the efficiency of the regulatory review process included training and education; training programmes for assessors; international workshops; external courses; in-house courses; on-the-job training; external speakers invited to the authority; induction training; sponsorship of postgraduate degrees; and placements and secondment in other regulatory authorities. Six authorities implement most of such measures. However, Burundi, Kenya and Uganda did not have a policy in place to invite external speakers to the authority; Burundi and Rwanda did not sponsor postgraduate degrees; Uganda reported that they do not host international workshops or conferences and along with Burundi and Rwanda, they do not make placements and secondments in other regulatory authorities.

In addition, it is now common practice in such regulatory authorities to implement reliance through either abridged or verification pathways for new active substances as well as biosimilars. This would be based on established memoranda of understanding (MOU) with stringent regulatory authorities such as the European Medicines Agency (EMA), United States Food and Drug Administration (US FDA), SwissMedic, Therapeutic Goods Administration (TGA) Australia, Health Canada and the Health Sciences Authority (HSA) Singapore. This has been clearly demonstrated in the recent publications by Danks et al. and McAuslane et al. (4, 5).

### 3.4 Part IV: quality decision-making practices

Ten quality decision-making practices were used to determine whether these authorities have measures in place to ensure that quality decisions are made using the data submitted during the review of applications: (1) Have a structured systematic approach to aid decision-making; (2) Assign clear roles and responsibilities; (3) Assign values and relative importance to decision criteria; (4) Evaluate both internal and external influences/biases; (5) Examine alternative solutions; (6) Consider uncertainty; (7) Re-evaluate as new information becomes available; (8) Perform impact analysis of the decision; (9) Ensure transparency and provide a record trail; (10) Effectively communicate the basis of the decision. Out of these ten quality decision-making practices, Kenya implemented four, Rwanda eight, Zanzibar three, Uganda five, Burundi eight and Tanzania implemented all the ten quality practices.

## 4 Discussion

The aim of this study was to evaluate GRevP in authorities participating in the EAC-MRH initiative and map strategies aligning with the African Medicines Agency. Comparing the similarities and differences of authorities in this region will assist them through information sharing to identify best practices in the process and documentation of the review procedures. It will also assess how these authorities build quality into their review processes. Ensuring standardisation, improvement in documentation, timeliness, predictability, consistency and high quality of reviews and review reports will entail efficient and effective GRevP in regulatory authorities. One of the key challenges faced by industry in applying for marketing authorisation has been the lack of detailed information (6, 7) on the regulatory procedures for applicants. This study which is similar to one conducted by Sithole et al. (3) for the South African Development Community (SADC) region will raise awareness within industry and applicants regarding the regulatory processes for each agency. This will enhance transparency and clarity on the application process, thereby leading to an increase in investments in medicines development and improved submission of applications to authorities in the region.

As a result of the participation of all the EAC authorities in the regional harmonisation initiative, they are now operating either as autonomous (3 authorities) or semi-autonomous authorities (4 authorities), improving the regulatory review processes of these authorities. One of the key challenges for regulatory systems strengthening in most countries in Africa is the absence of an autonomous NRAs mandated to regulate the market. In countries where regulatory functions are split among two or more authorities, there is usually duplication of effort, lapses in implementation, inconsistencies and inefficient use of limited resources. With autonomous authorities, efficiency and effectiveness can be ensured, as this governance structure enables the authority to focus on regulation (8). The African Union Model Law on medical products regulation (AU Model Law) provides for the establishment of autonomous NRAs for effective coordination and regulation of medical products in a country. However, article five of the AU Model Law recommends that agencies should be fully autonomous. This law was endorsed by the Heads of States and Governments in 2016 (9) whose objective is to promote collaboration across countries and provide an enabling environment for the manufacturing, testing and scaling up of essential and priority medical products in Africa. Five out of the six countries in the region have comprehensive legal frameworks, thereby providing a good foundation for effective regulation (10).

Challenges in human resource constraints are faced by all the agencies evaluated, and all had backlogs during the period of the study. Even though one of the strengths of the EAC-MRH initiative has been building the capacity of assessors in the region (6, 7), there is still a significant gap in terms of numbers of assessors in these agencies as per the results of this study. Strengthening of the harmonisation initiative, operationalisation of the AMA and reliance on well-resourced agencies by less resourced agencies are being proposed as some of the immediate interventions to address the challenge of limited resources (6, 7, 11). However, the results of this study demonstrate that the NRAs receiving the highest number of applications (Tanzania, Kenya, and Uganda) use both internal and external experts for the primary scientific assessment while the NRAs with less applications for review utilise only their internal technical authority staff for scientific assessments (12, 13).

One of the major challenges observed in this study is the recording of the timelines for each milestones achieved. These all vary amongst the NRAs in the regions, with most agencies not implementing a routine recording of timelines for key indicators such as timelines for validation. This comparative study will act as a baseline and will assist the NRAs to reflect on their key performance indicators as they build on the continuous monitoring of performance. Assessing the current situation will be a guide for making informed decisions on how to improve performance (3), as countries will learn from each other on how NRAs with similar resources conduct their reviews.

This study is also crucial for the EAC-MRH initiative, especially as it relies on country processes to register medical products that have been recommended by the joint review process. The current observation is that countries delay implementing the recommendations from the regional process. It is therefore important for the EAC-MRH program to revise its process to limit dependency on the country processes, which are already overwhelmed with the national workload. The understanding of country-specific requirements that follow an EAC-MRH positive opinion to address reasons for further delays in the approval process is key for the alignment to the AMA (6, 7).

### 4.1 Recommendations

The following are the recommendations emanating from this study.

1. **Independence of authorities** – Those authorities that are semi-autonomous should consider becoming fully autonomous and operating outside the administrative structure of their health ministry.
2. **Regulatory strengthening** – For those authorities that have limited resources, consideration should be given to engagement of external experts for the review of marketing authorisation applications.
3. **Sources of funding** – The funding structure of the authorities should be re-evaluated with respect to government and applicants’ fees, which should be commensurate with the effectiveness and efficiency of the authority operation.
4. **Communication with applicants** – The authorities should consider providing scientific advice to the applicants in order to streamline “clock stop, clock start” processes.
5. **Implementation of quality decision-making practices** – It is recommended that all the authorities implement the 10 Quality Decision-Making Practices underpinned by initiating appropriate structured training.
6. **Publication of the summary basis of approval** – In order to be transparent, it is recommended that the authorities make assessment reports available so that applicants might be aware of the basis on which an application was granted approval.

## 5 Conclusion

For the AMA to be successful and achieve its objectives, country regulatory processes need to be streamlined and differences in country requirements minimised. Six out of the seven authorities, with the exception of South Sudan, implemented all eight quality measures. It is noteworthy that the authorities included standard operating procedures for the guidance of the assessors, assessment templates, and a dedicated quality department.

A comparison of the transparency and communication parameters indicated that official guidelines were provided to assist the industry with their submission, facilitated tracking of the progress of applications was available and the authorities documented the approval of the products as well as their timelines. Furthermore, all six agencies reviewed the assessors and feedback. However, post-approval feedback to applicants on the quality of their submitted dossier, pre-submission scientific advice and the publication of the summary of grounds on which the approval was granted were formally implemented only by half of the agencies. It is imperative for countries to implement all good review practices in order to accelerate patients’ access to safe, high-quality, effective medical products when the AMA is established.

## Data Availability

The raw data supporting the conclusions of this article will be made available by the authors, without undue reservation.
